# SARS-CoV-2 (COVID-19) as a possible risk factor for neurodevelopmental disorders

**DOI:** 10.3389/fnins.2022.1021721

**Published:** 2022-12-16

**Authors:** Harikesh Dubey, Ravindra K. Sharma, Suraj Krishnan, Rebecca Knickmeyer

**Affiliations:** ^1^Division of Neuroengineering, Institute for Quantitative Health Sciences and Engineering, Michigan State University, East Lansing, MI, United States; ^2^Department of Physiology and Functional Genomics, University of Florida College of Medicine, Gainesville, FL, United States; ^3^Jacobi Medical Center, Albert Einstein College of Medicine, The Bronx, NY, United States; ^4^Department of Pediatrics and Human Development, Michigan State University, East Lansing, MI, United States

**Keywords:** SARS-CoV-2, COVID-19, HPA axis, preeclampsia, brain development, inflammation, pregnancy

## Abstract

Pregnant women constitute one of the most vulnerable populations to be affected by severe acute respiratory syndrome coronavirus 2 (SARS-CoV-2) infection, the cause of coronavirus disease 2019. SARS-CoV-2 infection during pregnancy could negatively impact fetal brain development via multiple mechanisms. Accumulating evidence indicates that mother to fetus transmission of SARS-CoV-2 does occur, albeit rarely. When it does occur, there is a potential for neuroinvasion via immune cells, retrograde axonal transport, and olfactory bulb and lymphatic pathways. In the absence of maternal to fetal transmission, there is still the potential for negative neurodevelopmental outcomes as a consequence of disrupted placental development and function leading to preeclampsia, preterm birth, and intrauterine growth restriction. In addition, maternal immune activation may lead to hypomyelination, microglial activation, white matter damage, and reduced neurogenesis in the developing fetus. Moreover, maternal immune activation can disrupt the maternal or fetal hypothalamic-pituitary-adrenal (HPA) axis leading to altered neurodevelopment. Finally, pro-inflammatory cytokines can potentially alter epigenetic processes within the developing brain. In this review, we address each of these potential mechanisms. We propose that SARS-CoV-2 could lead to neurodevelopmental disorders in a subset of pregnant women and that long-term studies are warranted.

## Introduction

In December of 2019 a novel virus, severe acute respiratory syndrome coronavirus 2 (SARS-CoV-2), caused a pneumonia outbreak in Wuhan City, Hubei Province in China. The disease caused by the virus was designated coronavirus disease 19 (COVID-19). SARS-CoV-2 expanded rapidly across the globe, and on March 11, 2020 the World Health Organization [WHO] (2022) declared COVID-19 a pandemic and global public health emergency. As of November 22, 2022 [WHO Coronavirus (COVID-19) Dashboard | WHO Coronavirus (COVID-19) Dashboard with Vaccination Data], 634 million cases have been detected worldwide. SARS-CoV-2 has already posed a great threat not only to the health of the people but also to the economy and healthcare system.

Severe acute respiratory syndrome coronavirus 2 (SARS-CoV-2) is an enveloped, positive stranded ribonucleic acid (RNA) virus of the family of Coronaviridae that causes respiratory and gastrointestinal infections ranging from mild, self-limiting conditions to more serious disorders such as viral pneumonia with systemic impairment (di Mascio et al., 2020). Unfortunately, pregnant women constitute one of the most vulnerable groups to be affected by this viral infection, due to anatomical, reproductive, endocrine, and immune changes (Zhao et al., 2020). With specific regard to the latter, immunological changes in pregnancy result in suppressed cell mediated immunity which would increase susceptibility to SARS-CoV-2 (Liu et al., 2020). In addition, pregnant women are more likely to have severe disease and ICU admissions compared to their non-pregnant counterparts after adjusting for age, underlying medical conditions, race, and ethnicity (Ellington et al., 2020; Sutton et al., 2020). As per a systematic review of over 11,000 pregnant women with suspected or confirmed COVID-19, the disease commonly manifests as fever (40%), cough (39%), dyspnea (19%), loss of taste (15%), myalgia (10%) and diarrhea (7%) (Allotey et al., 2020).

**FIGURE 1:**
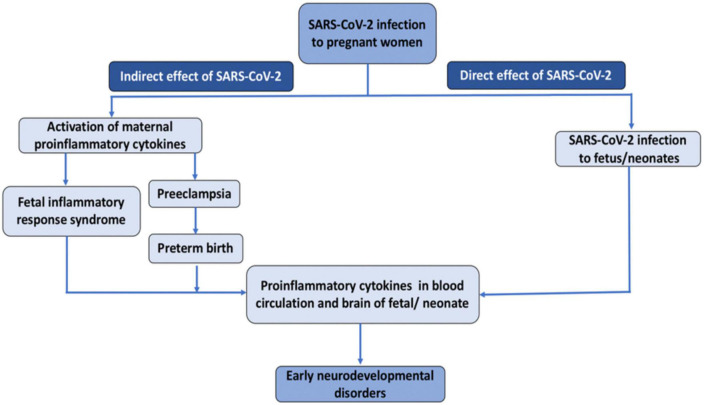
Direct and indirect effect of SARS-CoV-2 infection on fetal/neonatal brain development.

The novel SARS-CoV-2 infection could negatively impact fetal brain development in both direct and indirect ways (Figure 1) (Ellul et al., 2020). Regarding the direct route, an increasing number of case studies provide evidence for transplacental transmission of SARS-CoV-2, which could invade the central nervous system and disrupt brain development. Regarding indirect routes, SARS-CoV-2 could produce placental dysfunction, preeclampsia, and preterm birth, and trigger immune responses in the mother, which could, in turn affect the developing fetus. Interestingly, many of these routes involve the action of pro-inflammatory cytokines. Preclinical studies have revealed that inducing inflammation during the perinatal period produces long-term alterations in brain structure and function and a wealth of epidemiological studies have documented associations between infection-induced immune activation and offspring neuropsychiatric risk. In this review, we address each of these potential mechanisms and propose that SARS-CoV-2 could lead to neurodevelopmental disorders in a subset of pregnant women. We also review emerging empirical evidence supporting this hypothesis. This manuscript builds upon prior review articles on this topic such as (Shook et al., 2022), and (Figueiredo et al., 2021), which focused primarily on emerging evidence for transplacental transmission and the role of maternal immune activation (MIA), and (Kleeman et al., 2022), which focused primarily on epigenetic mechanisms in MIA. The current manuscript aims to be both comprehensive and concise. It provides a more detailed discussion of how altered levels of glucocorticoids in the context of SARS-CoV-2 could affect fetal neurodevelopment, and is the first, to our knowledge, to raise the possibility that SARS-CoV-2 induced hypocortisolism could be a risk factor for adverse neurodevelopmental outcomes. Finally, this review includes the most recent empirical studies on neurodevelopmental consequences of *in utero* exposure to SARS-CoV-2 including (Aldrete-Cortez et al., 2022; Hessami et al., 2022; Shuffrey et al., 2022).

## The direct route: Evidence of maternal transmission of SARS-CoV-2 infection to fetuses/neonates

Several viruses are known to be transmitted from pregnant women to their children and subsequently disrupt neurodevelopment. These are the TORCH pathogens. TORCH is an acronym standing for *Toxoplasma gondii*, Other infections, Rubella, human Cytomegalovirus (HCMV), and Herpes simplex viruses 1 and 2 (HSV-1 and HSV-2, respectively). “Other infections” include human immunodeficiency virus (HIV), syphilis, parvovirus B19 (fifth disease), varicella (chickenpox) and Zika (reviewed in Schwartz and Hyg, 2017). HCMV and HSV infections are the most common causes of neonatal morbidity worldwide (Looker et al., 2017; Marsico and Kimberlin, 2017) and in recent years Zika virus remains a threat for pregnant women (reviewed in Spitz, 2019). These TORCH pathogens can induce brain calcifications, major brain malformations including microcephaly, and neurodevelopmental disorders (Chen et al., 2021; Krenn et al., 2021). Consequently, one of the first questions we ought to ask when considering the adverse neurodevelopmental potential of SARS-CoV-2 is whether there is evidence of maternal transmission to the fetus or neonate.

Transmission of SARS-CoV-2 from mother to child could occur transplacentally, during labor and delivery, or in the early post-partum period. The possibility of vertical transmission of SARS-CoV-2 from mother to fetus *in utero* is currently a topic of widespread debate. The published literature is both sparse and contradictory, with some reports supporting direct *in utero* transmission (Bahadur et al., 2020; Dong et al., 2020; Patanè et al., 2020), and others suggesting little or no vertical transmission (Dashraath et al., 2020; Khan et al., 2020; Xiong et al., 2020; Zhu et al., 2020). The presence of SARS-CoV-2 either in amniotic fluid, placental samples, or infant nasopharyngeal swabs collected shortly after birth, represents compelling evidence for an *in utero* infection. Several case studies have been published documenting such evidence. Sisman et al. (2020) reported a case of congenital SARS-CoV-2 in an infant born through vaginal delivery to a COVID-19 positive mother. This report confirmed the intrauterine transmission of SARS-CoV-2 *via* presence of SARS-CoV-2 nucleocapsid protein and viral particles in placental syncytiotrophoblastic cells and nasopharyngeal samples of the infant (Sisman et al., 2020). Similarly, Patanè et al. (2020), demonstrated vertical transmission of SARS-CoV-2 from mother to fetus *in utero* as evidenced by the presence of SARS-CoV-2 RNA on the fetal side of placental tissues (Patanè et al., 2020). Transplacental transmission of SARS-CoV-2 is also supported by reports of caesarean delivery where strict neonatal isolation was implemented immediately after birth without delayed cord clamping or skin to skin contact. In one such case study, neonatal nasopharyngeal swabs were positive for SARS-CoV-2 RT-PCR test within 16 h of birth despite these precautions (Alzamora et al., 2020) and within 24 h in the other (Kirtsman et al., 2020). Perhaps the strongest early evidence supporting congenital infection was reported by Kirtsman et al. (2020). In this case, a woman with active SARS-CoV-2 delivered via caesarean delivery. The neonate had no contact with vaginal secretions or maternal skin. Artificial rupture of membranes was performed at operation, which was conducted with airborne, droplet, and contact precautions. The infant was immediately removed from the operative field, in a sterile fashion, to a resuscitator 2 m away in the same room. Never-the-less, neonatal nasopharyngeal swabs were SARS-CoV-2 positive by RT-PCR test on the day of birth as well as day 2 and day 7. Furthermore, placental micrographs revealed multiple areas of infiltration by inflammatory cells and extensive early infarction (Kirtsman et al., 2020). While, a number of researchers cautioned against treating early data as conclusive (Kimberlin and Stagno, 2020), evidence for transplacental transmission has continued to accumulate, though it appears to be a very rare event. In a study of 427 pregnant women from the UK admitted to hospital with SARS-CoV-2 infection, 12 of 265 infants tested positive, a rate of 5%, though only 6 of those did so within the first 12 h after birth (Knight et al., 2020). A recent systematic review and meta-analysis including data up to 3 August 2021 and including over 14,000 babies born to mothers with SARS-CoV-2 infection found about 2% of babies tested positive with 14 confirmed mother-to-child vertical transmission, seven of which occurred *in utero* (Allotey et al., 2022). A slightly smaller systematic review of 47 studies and over 900 neonates reported that slightly less than 1% had a confirmed or probable vertical transmission of infection (Jeganathan and Paul, 2022). Similarly, a recent “systemic review of systematic reviews” suggested that mother to child transmission was relatively rare with about 70% of cases attributable to environmental exposure and about 20% related to potential vertical transmission (Musa et al., 2021). Overall, it appears that transplacental transmission is possible, but rare.

## The direct route: The neuroinvasive potential of SARS-CoV-2

Severe acute respiratory syndrome coronavirus 2 (SARS-CoV-2) is similar in many ways to SARS-CoV, a virus identified in 2003, which is known have neuroinvasive potential (Ding et al., 2004; Xu et al., 2005), as evidenced by its presence in neural tissue from SARS autopsies (Gu et al., 2005). Studies indicate that the genomic sequence is similar between SARS-CoV-2 and SARS-CoV (Lu et al., 2020; Yu et al., 2020). It is particularly notable that the receptor-binding domain of SARS-CoV is structurally similar to SARS-CoV-2 (Lu et al., 2020). Hence, it is possible that SARS-CoV-2 follows the same path of neuroinvasiveness as SARS-CoV using the ACE2 receptor for cellular entry into the human brain. Several hypotheses have been put forth regarding possible mechanisms of SARS-CoV-2 mediated neural invasion including: (1) Transplacental transmission could induce viremia which would promote viral binding to the endothelial ACE2 receptors of the blood brain barrier (BBB) and subsequently entry into the central nervous system (CNS). Electron micrography on post-mortem brain biopsies revealed viral particles in the frontal cortex of a SARS-CoV-2 infected adult. The presence of particles in brain capillary endothelium and blebbing of viral-like particles coming in/out of the endothelial wall strongly suggested neuroinvasion through the BBB (Paniz-Mondolfi et al., 2020). (2) Cells of the immune system (macrophages and monocytes), which may express the ACE2 receptor, could act as a reservoir for dissemination into the CNS (Desforges et al., 2014). Further, infected immune cells (monocytes neutrophils and T cells) may disseminate into brain via various entry points including meninges, vasculatures, and the choroid plexus (Iadecola et al., 2020). (3) Neurons in the gut could carry the virus into the CNS via retrograde axonal transport (Esposito et al., 2020). (4) The virus could enter the CNS through the olfactory bulb. This possibility is strengthened by SARS-CoV-2 induced anosmia being a notable symptom during viral infection. Studies have shown expression of ACE2 receptors and other receptors that can facilitate SARS-CoV-2 binding in the olfactory epithelium (Fodoulian et al., 2020). This could play a role in neonatal infection during delivery through contact with vaginal secretions or soon after delivery through other means (physical or airborne). (5) Finally, the lymphatic pathway represents another possible route for neuroinvasion by the SARS-CoV-2 virus. The virus may directly enter the brain via olfactory/cervical lymphatic vessels (Bostancıklıoğlu, 2020).

One key question when considering the neuroinvasive potential of SARS-CoV-2 *in utero* is whether the fetal brain expresses cellular components that interact with the spike protein of SARS-CoV-2. Using publicly available RNA sequencing datasets, Varma et al. (2021) revealed that while *ACE2* mRNA is expressed at relatively low levels in the fetal brain, other spike protein interactors including *FURIN*, *ZDHHC5, GOLGA7*, and *ATP1A1* are highly expressed, especially in neurons. These proteins may play key roles in SARS-CoV-2 fetal brain pathogenesis, especially during the 2nd and 3rd trimesters of pregnancy (Varma et al., 2021).

## The indirect route: SARS-CoV-2 effects on the placenta and ensuing complications

Even in the absence of direct transmission of a pathogen from mother to child, infections can disrupt neurodevelopment in indirect ways. For example, the H1N1 influenza virus is not teratogenic, but severe infections were associated with elevated risks for adverse infant outcomes, such as preterm birth, which have neurodevelopmental consequences [Maternal and Infant Outcomes Among Severely Ill Pregnant and Postpartum Women with 2009 Pandemic Influenza A (H1N1) — United States, April 2009–August 2010; Newsome et al., 2019]. In this section we discuss emerging evidence that SARS-CoV2 impacts placental functioning and how this could lead to altered neurodevelopment.

Severe acute respiratory syndrome coronavirus 2 (SARS-CoV-2) binding ACE2 receptors are highly expressed in placental tissues. SARS-CoV-2 infected pregnant women have placental inflammatory signs along with systemic maternal inflammation (Vivanti et al., 2020), which can lead to placental microvascular dysfunction. This may present clinically as preeclampsia or preeclampsia-like features, fetal distress, intrauterine growth restriction, and/or or preterm labor depending on gestational age at time of SARS-CoV-2 infection (Mulvey et al., 2020).

To understand the impact of COVID-19 infection on the placenta, a brief review of the salient aspects of the renin–angiotensin system (RAS) axis in the formation of a well perfused placental vascular bed may be helpful. In the maternal portion of the human placenta, which is derived from the maternal stromal cells, ACE2 is highly expressed in the invading and intravascular trophoblast and in decidual cells. ACE2 is also found in arterial and venous endothelium and smooth muscle of the umbilical cord (Valdés et al., 2006). Levels of ACE2 vary temporally depending on gestational age in both humans and rodents (Valdés et al., 2006; Ghadhanfar et al., 2017). The various components of RAS-Ang II, ACE2, and Ang-(1-7) function mainly to regulate blood pressure and fetal development. Ang II stimulates trophoblast invasion in rat and human cells (Hering et al., 2010). Ang-(1-7) and ACE2 may act as local autocrine/paracrine regulators in the early (angiogenesis, apoptosis, and growth) and late (uteroplacental blood flow) events of pregnancy (Neves et al., 2008). ACE2 hydrolyzes Ang II into Ang-(1-7), and Ang I into Ang-(1-9), which is quickly converted to Ang-(1-7), thereby controlling the blood pressure and hydro-salinity balance of pregnant women (Pringle et al., 2011).

Preeclampsia is a serious pregnancy complication which typically begins after the 20^th^ week of gestation and manifests as high blood pressure and proteinuria. The exact cause of preeclampsia is not known, but it is generally believed to arise due to improper functioning of the placenta. Placental vascular anomalies and inflammation are often observed in women affected by preeclampsia (Harmon et al., 2016; Ramos et al., 2017). Brosnihan et al. (2004), reported that pre-eclamptic women presented with suppressed plasma Ang-(1-7) levels when compared with normal pregnancy subjects (Brosnihan et al., 2004). Furthermore, high expression of Ang II in the placental villus during preeclampsia can cause decreased blood flow and nutrition supply to the fetus (Shibata et al., 2006; Anton and Brosnihan, 2008; Anton et al., 2009). An observational study published early in the pandemic suggested that prevalence of preeclampsia is remarkably higher in SARS-CoV-2 infected pregnant women, with five out of eight infected pregnant women admitted to the intensive care unit having preeclampsia like syndrome (Mendoza et al., 2020), which may lead to higher occurrence of preterm birth (Yan et al., 2020). A case report by Hosier and colleagues might be an example of placental infection with SARS-CoV-2 or manifestation of SARS-CoV-2 induced cytokine release or both presenting as severe early-onset preeclampsia. The patient, who had a history of gestational hypertension in a prior pregnancy, but normal blood pressure during early pregnancy and normal baseline preeclampsia evaluation, presented acutely at 22-weeks’ gestation with features mimicking preeclampsia with Disseminated Intravascular Coagulation (DIC) and fever. SARS-CoV-2 RNA was positive in a nasopharyngeal swab. Patient’s thrombocytopenia and hypofibrinogenemia were more severe than what would have been expected from SARS-CoV-2 alone. Placental pathology post termination of the pregnancy revealed SARS-CoV-2 localized to the synciotiotrophoblast layer and the intervillous invasion of macrophages (intervillositis) (Hosier et al., 2020). A study by Mulvey et al. (2020), on placenta (after term delivery) of COVID-19 infected pregnant women found fetal vascular malperfusion due to focal avascular villi and thrombi in large fetal vessels (Mulvey et al., 2020). Vivanti et al. (2020) also reported a case of delivery at 35 weeks through cesarean section following fetal distress indicated by category 3 fetal heart rate tracing, and RT-PCR in the placenta was positive for SARS-CoV-2. The mother who was having an uneventful pregnancy until diagnosis of COVID-19, without any severe or critical presentation of the infection, had thrombocytopenia, lymphopenia, elevated acute phase reactants, and abnormalities in coagulation cascade on admission. Three days after hospitalization, without any deterioration of maternal status, a category 3 fetal heart tracing was observed, representing fetal compromise likely due to uteroplacental insufficiency for which delivery through cesarean section was performed (Vivanti et al., 2020). This strongly indicates that SARS-CoV-2 can cause uteroplacental dysfunction and can induce a preeclampsia like picture either due to direct placental invasion or through induction of excess cytokine release in the mother or both. However, one must acknowledge that much of the empirical evidence for this hypothesis comes from case studies, which may not generalize to all SARS-CoV-2 infected mothers. Case studies may also be subject to researcher bias and do not allow the production of quantifiable risk estimates. A recent systematic review of cardiovascular complications among pregnant women with COVID-19 found substantial variance in estimates across studies with some reporting rates of preeclampsia as high as 69% and others as low as 0.5% (Yaghoobpoor et al., 2022).

**FIGURE 2:**
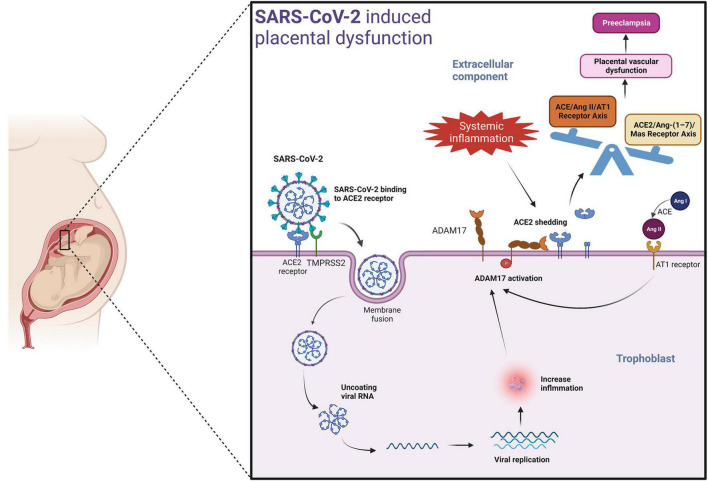
Mechanism involved in SARS-CoV-2 induced placental dysfunction.

A potential mechanism by which SARS-CoV-2 could induce placental dysfunction and preeclampsia is illustrated in Figure 2. The viral spike protein of SARS-CoV-2 facilitates binding to the ACE2 receptor. When viremia occurs in the mother during severe SARS-CoV-2 infection, the virus may invade the placenta. The viral spike protein of SARS-CoV-2 after binding with ACE2 receptor, enters the placental trophoblast with the help of protease-mediated cleavage (TMPRSS2 or cathepsin L) of the S protein subunit- S2. This internalization of the virus along with the ACE2 receptor into the placental trophoblast may increase the activity of ADAM17 (a matrix metalloproteinase) (reviewed in Schreiber et al., 2021; Jackson et al., 2022), as seen in previous SARS-CoV infection (Haga et al., 2008). ADAM17 up-regulation leads to proteolytic cleavage of the ACE2 ectodomain (Lambert et al., 2005; Heurich et al., 2014), which results in reduced membrane ACE2. The resulting imbalance in AngII/ACE2 interaction may result in hypertension of pregnancy, pre-eclampsia, or eclampsia in susceptible mothers. Of note, systemic inflammation and oxidative stress induced by SARS-CoV-2 infection may also lead to increased ADAM17 expression in the placental trophoblasts promoting the development of preeclampsia in susceptible women even in the absence of direct placental infection by SARS-CoV-2 (Gooz, 2010) (Figure 2).

Even in the absence of preeclampsia, SARS-CoV-2 infection in the early stages of pregnancy can cause fetal growth restriction (FGR)/intrauterine growth restriction (IUGR) (Allotey et al., 2020), which is itself a risk factor for abnormal postnatal neurodevelopment in babies (Adams Waldorf and McAdams, 2013; Dang et al., 2020). IUGR influences the overall growth of fetus and is accompanied by reduced total brain volume, which may cause cognitive (Hartkopf et al., 2018; reviewed in Miller et al., 2016) and motor regulation deficits (Dubois et al., 2008; Miller et al., 2016; Hartkopf et al., 2018; reviewed in Miller et al., 2016), and as well as some neurodevelopmental disorders. More specifically, reduced brain volumes have been observed in schizophrenia and bipolar disorder (Wright et al., 2000; McDonald et al., 2004; Arnone et al., 2009; Ellison-Wright and Bullmore, 2010; Haijma et al., 2013; Hibar et al., 2016, 2018; van Erp et al., 2016, 2018) and in ADHD (Boedhoe et al., 2020).

## The indirect route: SARS-CoV-2 induced inflammation may disrupt offspring neurodevelopment

Epidemiological studies indicate a strong correlation between maternal viral infection and neuropsychiatric disorders in offspring, especially for ASD and schizophrenia (reviewed in Estes and McAllister, 2016). For ASD, this includes a large-scale registry-based study in Denmark which revealed that severe viral infections (requiring hospitalization) during the first trimester of pregnancy are associated with increased risk of ASDs in offspring (Atladóttir et al., 2010) and a cohort study conducted in Finland which suggested that early stage increases in gestational CRP due to prenatal infection increases risk of ASD in children by 43% (Brown et al., 2014). A meta-analysis of 15 studies, conducted in 2016 and including over 40,000 ASD cases, reported an OR of 1.13 for the association between maternal infection during pregnancy and increased risk of ASD in offspring [95% confidence interval (CI): 1.03–1.23], with risk being moderated by the severity of infection, type of infectious agent, time of infectious exposure, and site of infection. Greater severity of infection (indexed by hospitalization) was associated with higher risk. Bacterial infections appeared to confer greater risk than viral infections and genitourinary and skin infections appeared to confer higher risk than gastrointestinal or respiratory infections. With specific regard to infection timing, second trimester exposures conferred the greatest risk, followed by first trimester exposures, and third trimester exposures had minimal effects (Jiang et al., 2016). This differs from a recent meta-analysis evaluating the impact of maternal fever on neurodevelopmental disorders in general (includes ASD, ADHD, Developmental Delay, and Developmental Coordination Disorder) which suggested first trimester exposures were most detrimental. Regarding schizophrenia, since Mednick et al. (1988) published their seminal, ecological study revealing increased risk of schizophrenia in pregnant women exposed to the 1957 influenza A epidemic in Helsinki, evidence linking schizophrenia to maternal infection has accumulated and includes (Mednick et al., 1988; Barr et al., 1990; Susser et al., 2000; Brown et al., 2001, 2004a,b,^2005^; Buka et al., 2001; Mortensen et al., 2007). A recent meta-analysis of seven cohort studies reported that maternal infection during gestation increased the risk of non-affective psychosis with a relative risk (RR) of 1.28 (95% CI:1.05-1.57) (Saatci et al., 2021). Relative risk was even high for schizophrenia (1.63) with the strongest effects observed in the second trimester. Several studies have highlighted the second trimester as the time of greatest risk (Brown et al., 2000; Nielsen et al., 2013), which may indicate an impact of infection on gestational neurogenesis, which peaks during this period (Stiles and Jernigan, 2010). However, another study reported that the risk of schizophrenia was increased 7-fold for influenza exposure during the first trimester with no increased risk of schizophrenia for exposure during the second or third trimester (Brown et al., 2004a). Another study indicating that first trimester prenatal exposures may increase risk for schizophrenia in offspring is (Clarke et al., 2009). Despite these inconsistencies regarding timing of exposure, the general idea that gestational infection predisposes individuals to schizophrenia is widely accepted. This is not to say there are no controversies in the literature. For example, Selten et al. (2010) published a meta-analysis challenging earlier studies linking the 1957 influenza pandemic to schizophrenia (Selten et al., 2010). In addition, even large population-based studies can suffer from methodological problems such as misclassification of exposure and genetic confounding. Karlsson and Dalman (2020) argue that infections in general appear to have a much smaller effect on schizophrenia risk compared to specific exposures such as *Toxoplasma gondii* (Karlsson and Dalman, 2020). Of particular relevance to SARS-CoV-2, research suggests that second trimester respiratory infections are a risk factor for schizophrenia spectrum disorders (Brown et al., 2000). There are also epidemiological studies linking maternal infections to ADHD (Pineda et al., 2007; Mann and McDermott, 2011; Silva et al., 2014) and mood disorders (see Simanek and Meier, 2015 for review), but as the evidence is more limited (for the former) and ambiguous (for the latter), we do not provide additional details here. Activation of the pregnant mother’s immune system in response to infection is thought to be the primary mechanism responsible for these associations, a hypothesis that is supported by a substantial body of preclinical research (reviewed in Boksa, 2010; Careaga et al., 2017).

Like other viral infections, SARS-CoV-2 can trigger systemic inflammation during pregnancy in both mother and fetus. ACE2 receptors are expressed widely in the mouth, tongue, respiratory tract, lung, heart, kidney, gut, endothelium, and in other tissues like placental tissues (Deverman and Patterson, 2009). Binding of ACE2 located on the surface of the target cells with the receptor-binding domain of SARS-CoV-2 results in endocytosis and translocation of both viruses and ACE2 into the endosomes located in the cell. Inside the cell, it replicates and induces cytotoxicity. The damaged host cell undergoes pyroptosis and releases damage-associated molecular patterns resulting in the initial inflammatory response.

As noted in the introduction, pregnant women are more likely to experience severe SARS-CoV-2 infection and ICU admissions compared to their non-pregnant counterparts, and COVID-19 can induce a systemic inflammatory disorder. With increasing severity of the infection, higher levels of circulating cytokines and other inflammatory biomarkers like IL6, IL-1β, TNFα, C-reactive protein (CRP) and D-dimer occurs (Smith et al., 2007; Deverman and Patterson, 2009; Wu et al., 2017; Zupan et al., 2017). These proteins attract monocytes, macrophages, and T-cells to the site of infection, promoting further inflammation and establishing a pro-inflammatory feedback loop. In addition, non-neutralizing antibodies produced by B-cells may enhance SARS-CoV-2 infection through antibody-dependent enhancement, further exacerbating organ damage (Deverman and Patterson, 2009). The resulting cytokine storm circulates to other organs, leading to multi-organ damage.

**FIGURE 3:**
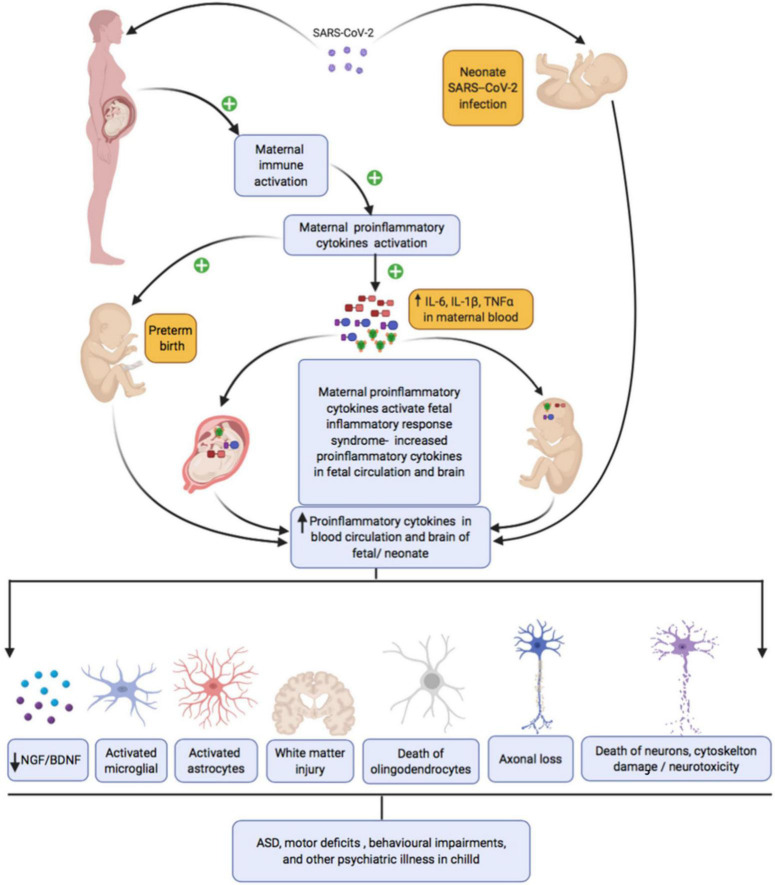
Maternal SARS-CoV-2 infection, inflammation, and subsequent fetal/neonatal brain development.

Even in the absence of a cytokine storm, maternal immune activation during COVID-19 along with proinflammatory changes in the placental vascular bed could potentially activate interleukin (IL-6) signaling in the syncitiotrophoblast layer. Furthermore, increased cytokines and complement factors in the maternal environment can bleed over into the fetus, altering neurodevelopment. Pro-inflammatory cytokines including IL-6, IL-1β, and TNF-α have a molecular mass of about 50kDa and can easily cross the placental barrier, passing from mother to fetus (Zaretsky et al., 2004; Aaltonen et al., 2005; Smith et al., 2007; Deverman and Patterson, 2009; Ratnayake et al., 2013; Wu et al., 2017; Zupan et al., 2017). All neural and non-neural cell types within the developing CNS use cytokines for paracrine and autocrine signaling. Thus, maternal immune activation secondary to maternal infection can disrupt brain development in multiple ways, which we review briefly in the following paragraphs and summarize in Figure 3. Most of the studies discussed in the ensuing paragraphs used rodent models of maternal immune infection (MIA). Multiple models of MIA exist including prenatal administration of immunogenic liposaccharides (LPS), transmembrane protein toll-like receptor (TLR) 4, or polyriboinosinic–polyribocytidilic acid [Poly(I:C)]. The specific models used are noted throughout.

First, maternal immune activation can trigger periventricular white matter damage. White matter damage has been observed in the context of many different prenatal infections in human newborns and in animal models [Yoon et al., 1997 (rabbit, E. coli infection); Dammann et al., 1999; reviewed in Malaeb and Dammann, 2009], and in a newborn baby following transplacental transmission of SARS-CoV2 infection (Vivanti et al., 2020). These observations partly reflect associations between prenatal infection and preterm delivery, which is a well-established risk factor for intraventricular hemorrhage, neonatal white matter damage, and subsequent cerebral palsy. However, rodent studies confirm that white matter injury in offspring can be induced by intrauterine maternal infection in the absence of preterm birth [Wang et al., 2007; Zhan et al., 2021 (LPS)]. Mechanisms of injury may include both direct effects of pro-inflammatory cytokines on oligodendrocytes and axons and indirect effects via activation of microglia (reviewed in Robinson, 2005; Burd et al., 2012). Microglial cells enter the human brain as early as 4 weeks of gestation and accumulate in the prospective white matter of the corona radiata between weeks 19 and 24 (Monier et al., 2007). When activated, microglia cause localized neuroinflammation and injury [ Monier et al., 2007; reviewed in Burd et al., 2012]. Once activated, the microglia may continue to remain activated into infancy or early childhood, resulting in sustained production of pro-inflammatory cytokines, oxidative and nitrosative products, and excitotoxic metabolites such as glutamate and quinolinic acid, all of which can injure oligodendrocytes. Specific pro-inflammatory cytokines that have been linked to oligodendrocyte injury include IL-1β which impairs myelination by reducing the number of developing oligodendrocytes when injected into the cerebrum of rat pups (Cai et al., 2004) and TNF-α which induces death of human oligodendrocyte cells by activation of apoptosis-inducing factor (Yoon et al., 1997; Saliba and Henrot, 2001), With regard to axonal development, Makinodan et al. (2008) report that juvenile mice had reduced axonal diameters in the hippocampus following maternal immune activation [poly(I:C)], a phenotype that normalized by adulthood.

Second, maternal immune activation can influence developmental neurogenesis and neurodifferentiation. In general, hyperactivation of the immune response is thought to impair survival and differentiation of neural progenitors (Borsini et al., 2015; Kim et al., 2016) by attenuating the production of neurotrophic factors including brain-derived neurotrophic factor (BDNF), nerve growth factor (NGF), platelet-derived growth factors (PDGF), and neurotrophins (NT-3,4). Much of this evidence is based on *in vitro* models, which are though to best model human midgestational neurogenesis. However, *in vitro* models cannot fully capture the impact of infection on a pregnant mother. Furthermore, different cytokines induce different effects in *in vitro* models – some positive and some negative. For example, IL-1β and TNF-α reduce neurogenesis of fetal hippocampal neural progenitor cells (NPCs) (Johansson et al., 2008; Zunszain et al., 2012; Chen et al., 2013), while IL-6 was reported to increase neurogenesis in human hippocampal NPCs (Johansson et al., 2008). Very few animal studies have directly assessed the impact of MIA on developmental neurogenesis. One recent study examined Ki67 + /Nestin + and Tbr2 + neural progenitor cells in the subventricular zone (SVZ) of neonatal mice following mid-gestation MIA (LPS) and reported robust increases (Loayza et al., 2022). In a similar manner, significant increases in the proportion of Pax6-positive neural progenitor cells and Pax6/Tbr2 double-positive cells have been observed in mouse fetal brains 24 h after poly(I:C) injection (Tsukada et al., 2021). This contrasts with the findings of Canales et al. (2021) who reported evidence of overall decreased neurogenesis by E17.5, following poly(I:C) injection at E12.5 (Canales et al., 2021) and with Tsukada et al. (2015) who reported that mid-gestatational MI (Poly:I:C) impairs neurogenesis in the cerebellum. There is a rich body of literature demonstrating that pro-inflammatory cytokines disrupt neurogenesis in the adult rodent hippocampus [see (Kim et al., 2016) for review] and several MIA studies examined adult hippocampal neurogenesis. Mid-gestation MIA (LPS) suppressed hippocampal neurogenesis in adult rat (Okano et al., 2022), as did late gestation treatment with Poly(I:C) (Zhao et al., 2019), while suppression of maternal IL-6 enhanced it (Mouihate and Kalakh, 2021). Finally, defective neurogenesis in the subventricular zone (SVZ)-olfactory bulb (OB) pathway has been reported following early gestational exposure to Poly:I:C in mouse (Liu et al., 2013). The mechanisms by which MIA primes dysfunction in the unique hippocampal pool of neural stem/progenitor cells in adulthood remains to be fully elucidated (Couch et al., 2021). Furthermore, effects of specific cytokines on neurogenesis and differentiation may vary based on brain region, species, and developmental stage.

Finally, pro-inflammatory cytokines can promote cytoskeletal damage and neural apoptosis. Astrocytes may play a key role in this process. MIA (Poly:I:C) induces a hypertrophied morphology and intense GFAP immunoreactivity in astrocytes in the hippocampus that persist at least until weaning (Patro et al., 2013), with upregulation of GFAP detectable in adulthood following LPS (Berkiks et al., 2019). Hypertrophied morphology and upregulation of GFAP indicate astrocytic activation and astrocytes produce reactive oxygen species (ROS) including nitric oxide (NO), which are neurotoxic. Activated microglia also produce ROS, as discussed previously, which could damage neurons as well as glia. Increased oxidative stress has been observed in the hippocampus and cerebral cortex of adult rats exposed to LPS MIA (Cieślik et al., 2020, 2021). In Cieślik et al. (2021), oxidative stress did not appear to result from activated microglia but was accompanied by evidence of mitochondrial dysfunction (Cieślik et al., 2021), which has also been linked to oxidative damage in mouse models of autism spectrum disorder (Yui et al., 2015). Cieślik et al. (2021) also observed abnormal phosphorylation and dysfunction of MAPT, which is involved in assembling and stabilizing microtubules, which make up the cytoskeleton (Cieślik et al., 2021). Increased neural apoptosis appears to be linked to late-gestational MIA, rather than mid-gestation MIA (Meyer et al., 2006) [poly(I:C), mice] and appears to arise due to the interactive effect of multiple cytokines (Matelski et al., 2021) (*in vitro* model).

We end this section by noting that animal models of MIA can also help address questions about how timing of infection relates to neurodevelopmental sequelae. For example, Nakamura et al. (2022) recently reported that early gestational exposure to MIA [poly(I:C)] disrupted working memory and reduced perseverative behavior in female offspring while late gestational exposure induced male-specific deficits in working memory and reversal learning (Nakamura et al., 2022). Guma et al. (2022) have reported that early gestational exposure to MIA [poly(I:C)] induces profound reductions in certain regions of the embryonic brain, likely through increased apoptosis, while late gestational exposure induced volume expansions, possibly due to acute inflammatory responses (Guma et al., 2022). The same group has also presented timing of exposure specific effects on neonatal mice brain volumes in regions of the amygdala, hippocampus, entorhinal cortex, striatum, and periaqueductal gray matter and reported that neonatal communication abilities, indexed by ultrasonic vocalizations, are reduced following early, but not late exposure (Guma et al., 2021b). Early exposure also appears to produce more profound effects on anxiety-like, stereotypic, and sensorimotor gating behaviors, measured in adolescence, than late exposure, changes that are accompanied by transcriptional alteration in genes linked to inflammation and autistic behaviors (Guma et al., 2021a).

## A key role for IL-6 in abnormal neurodevelopment following maternal immune activation

Maternal immune activation (MIA) is accompanied by increased levels of multiple pro-inflammatory cytokines. However, IL-6 appears to play an especially important role in mediating the impact of maternal infection on offspring neurodevelopment (Smith et al., 2007). This was demonstrated by Smith et al. (2007) via an elegant series of rodent experiments. First, they showed that administration of IL-6 during pregnancy was sufficient to induce prepulse inhibition (PPI) and latent inhibition (LI) deficits in adult offspring, while administration of IFNγ was not. Second, they demonstrated that administration of an IL-6-neutralizing antibody during MIA [poly(I:C)] rescued deficits in PPI and IL and normalized exploratory and social behavior. Next, they showed that IL-6 knock-out mice failed to exhibit deficit in PPI, social interaction, or exploratory behavior following MIA. Finally, they demonstrated that administration of an IL-6-neutralizing antibody during MIA also normalized gene expression differences in the brains of offspring (Smith et al., 2007). Hence, IL-6 emerged as the main driving factor through which MIA causes long term behavioral changes in offspring (Smith et al., 2007).

The importance of IL-6 in human brain development has been demonstrated via neuroimaging studies of infants and children. Specifically, Rudolph et al. (2018) reported associations between maternal IL-6 levels and neonatal functional brain connectivity, with the salience, dorsal attention, and subcortical networks being most extensively involved. Furthermore, these associations may explain associations between maternal IL-6 and offspring working memory performance at 2 years of age in the same sample (Rudolph et al., 2018). Effects of IL-6 on the salience network were also reported by Spann et al. (2018). Also, MRI data of infants (*n* = 30) shows that higher levels of IL-6 during pregnancy may lead to disruption in frontolimbic white matter and cognitive development (Rasmussen et al., 2019). Furthermore, children born to women (*n* = 86) with high IL-6 levels during early pregnancy showed larger right amygdala volumes and stronger bilateral amygdala connectivity to other parts of brain including fusiform, somatosensory cortex and thalamus (for sensory processing and integration), anterior insula (for salience detection), caudate and parahippocampal gyrus (for learning and memory) at 24 months age. Moreover, volume of the right amygdala and stronger left amygdala connectivity mediated associations between maternal IL-6 and compromised impulse control in offspring (Graham et al., 2018). While these studies are relevant to the issue at hand, it is important to note that the sample sizes are relatively small. A recent study by Marek et al. (2022), suggests that rigorous and reproducible associations between brain structure or function and complex cognitive or behavioral data may require thousands of individuals (Marek et al., 2022). In addition to being insufficiently powered, small sample sizes are vulnerable to sampling variability, inflated effect sizes, high statistical error rates, and poor reproducibility.

Various mechanisms have been proposed to explain how IL-6 induces abnormal neurodevelopment (Boulanger-Bertolus et al., 2018). Many of these mechanisms highlight the placenta as a key organ in this pathophysiological process. Knockout of the trophoblastic IL-6 receptor in mice prevents cerebellar neuropathology and behavioral impairments following MIA [poly(I:C)] and attenuates immune responses in the fetal brain (Wu et al., 2017). This suggests that placental IL-6 signaling, specifically in the trophoblast, is required for MIA-induced acute immune activation in the fetal brain and subsequent detrimental effects on offspring neurodevelopment, at least in rodents. The authors of this study proposed three different ways placental IL-6 signaling might impact the fetal brain. First, they proposed that the placenta may initiate a feed-forward cycle of IL-6 induction in the embryo. Second, they proposed effects of placental IL-6 signaling on the fetal brain might be mediated by changes in placental hormones including prolactin and corticotrophin-releasing factor (CRF). Finally, they suggested that IL-6 might induce trophoblasts to produce factors that increase vascular permeability in the placenta, thereby altering the metabolic and nutritional environment of the fetus. An earlier study by the same group revealed another placental hormone system disrupted by poly(I:C) MIA – the growth hormone-insulin-like growth factor (GH-IGF) axis. Levels of growth hormone (GH), insulin like growth factor 1 (IGF1), and insulin like growth factor binding protein 3 (IGFBP3) levels were all reduced following MIA (Hsiao and Patterson, 2011). More recently, Monteiro et al. (2022) reported that mid-pregnancy MIA [poly(I:C)] alters expression of placental ATP-Binding Cassette (ABC) efflux transporters, which transport a variety of substances including cholesterol, drugs, xenobiotics, and cytokines across the placental barrier (Monteiro et al., 2022). While this data is clearly of interest, it should be noted that there are substantial differences between the most frequently used rodent models and human placentas, which make it difficult to extrapolate directly from mice and rats to human. Schmidt et al. (2015) and Carter (2020) review several key differences in anatomy including (1) in human placenta maternal blood perfuses the intervillous space, while in mice and rats exchange of material is between fetal and maternal capillaries, (2) humans do not have an inverted yolk sac placenta in addition to the chorioallantoic placenta, while mice and rats do, (3) mice and rats have trichorial placentas while humans have monochorial placentas, and (4) in humans there is deep interstitial and endovascular invasion of trophoblast cells into the inner third of the human myometrium, while in mice the invasion is restricted to the decidua basalis (Schmidt et al., 2015; Carter, 2020). The latter difference is particularly problematic when studying preeclampsia. There are also important species differences in placental endocrinology, molecular features, and immune responses. For example, the human placenta can actively transport protective immunoglobulin IgG antibodies to the fetus during gestation, while rodents do not transport IgG as efficiently and mice acquire maternal IgG antibodies via yolk sac–derived cells and after birth via suckling (reviewed in Ander et al., 2019). In addition, the human placenta secrets primate-specific antiviral microRNAs (miRNAs) from a cluster on chromosome 19 (C19MC) from syncytiotrophoblast layer that broadly restrict viral infections while mouse placenta uses interferons to restrict viral infections (Ander et al., 2019). To overcome this translational challenge, additional studies using *in vitro* models and alternative animal models are needed (Schmidt et al., 2015; Ander et al., 2019; Carter, 2020).

## Maternal infection and fetal hypothalamic-pituitary-adrenal axis modulation

It is well established that viral infection increases the production of proinflammatory cytokines which in turn activate the HPA axis, resulting in increased glucocorticoid production (reviewed in Silverman et al., 2005; Raony et al., 2020). When the maternal HPA axis is activated, levels of glucocorticoids in maternal blood increase. Glucocorticoids can cross the placental barrier thereby increasing fetal glucocorticoid levels. Also, maternal cytokines can cross the placenta and activate the fetal HPA axis and stimulate the release of corticotrophin releasing hormone (CRH). This subsequently would stimulate secretion of adrenocorticotrophic hormone (ACTH) from the fetal anterior pituitary and glucocorticoids from the fetal adrenals (reviewed in Seckl, 2004; Ratnayake et al., 2013). Glucocorticoids play a central role in the fetal programming of HPA function (reviewed in Kapoor et al., 2006). Exposure to high levels of glucocorticoids *in utero* can manifest as disrupted HPA axis reactivity in later life and may underlie cognitive deficits and addictive behaviors in childhood and adulthood (reviewed in Waffarn and Davis, 2012; Moisiadis and Matthews, 2014; Granja et al., 2021).

In a recent review article, Granja et al. (2021) proposed a possible mechanism by which SARS-CoV-2 infection in a pregnant woman may disrupt fetal brain development via interference with the HPA axis. They explain that during a normal pregnancy, levels of 11β-HSD2 (glucocorticoid inactivating hormone) increase to ensure the appropriate exposure of glucocorticoids to the fetus. At the same time progesterone levels are also increasing to counter the cytokine balance toward an anti-inflammatory profile at the maternal-fetal interface (Granja et al., 2021). They hypothesize that viral infection (e.g., SARS-CoV-2 infection) may disrupt placental 11β-HSD2 expression resulting in increased exposure of the fetus to glucocorticoids (Granja et al., 2021). We are unaware of any studies directly testing Granja et al’s hypothesis, but there is a growing body of literature on the immune environment of the human placenta during COVID-19 infection. Lu-Culligan et al. (2021) reported robust inflammatory responses in placenta tissue from third trimester COVID-19 infections including increased expression of pro-inflammatory genes and chemokines, revealed by single-cell transcriptomic profiling (Lu-Culligan et al., 2021). In contrast, Juttukonda et al. (2022) have reported that while decidual tissue from individuals with third trimester COVID-19 infections have increased macrophages, NK cells, and T cells, levels of IL-8 are reduced compared to controls and levels of IFN-γ, IL-1β, IL-6, IL-10, and TNF do not differ (Juttukonda et al., 2022). In the same study, decidual tissue from individuals with second trimester infections showed a significant decrease in IL-6, IL-8, IL-10, and TNF-α and no change in abundance for IL-1β or IFN-γ (Juttukonda et al., 2022). Bordt et al. (2021) reported increased levels of IFN-α, IFN-γ, and IL-10 in placentas from individuals with third trimester infections, but only in males (Bordt et al., 2021). Thus, there is still much to be done in terms of understanding how maternal COVID-19 impacts inflammatory profiles of the placenta and a dearth of studies on how this might impact glucocorticoids.

Maternal glucocorticoids can reach fetal brain and bind with glucocorticoid receptors (GR) to exert detrimental effects (reviewed in Miranda and Sousa, 2018). In fact, disrupted placental 11β-HSD2 expression, may lead to abnormal glucocorticoid receptor (GR) expression in hippocampus and amygdala, leading to a hyperreactive HPA axis and increased anxiety-like behaviors in adult rat offspring (Welberg et al., 2000). Furthermore, higher glucocorticoid exposure to the fetus increases inducible nerve growth factor A and activates transcriptional activity thereby disrupting fetal brain development (Andrews et al., 2004) (guinea pig). In addition to these molecular changes, fetal exposure to high levels of glucocorticoids also impacts neurogenesis in both rodent and human *in vitro* models and changes hippocampal structure (Gould et al., 1992; Lemaire et al., 2000; Provençal et al., 2020). Similarly, higher levels of glucocorticoid may alter microglial function (reviewed in Walker et al., 2013), disrupting synaptogenesis, neurogenesis, synaptic pruning, axonal growth, myelination and astrocyte maturation (reviewed in Harry and Kraft, 2012; Schafer and Stevens, 2015).

**FIGURE 4:**
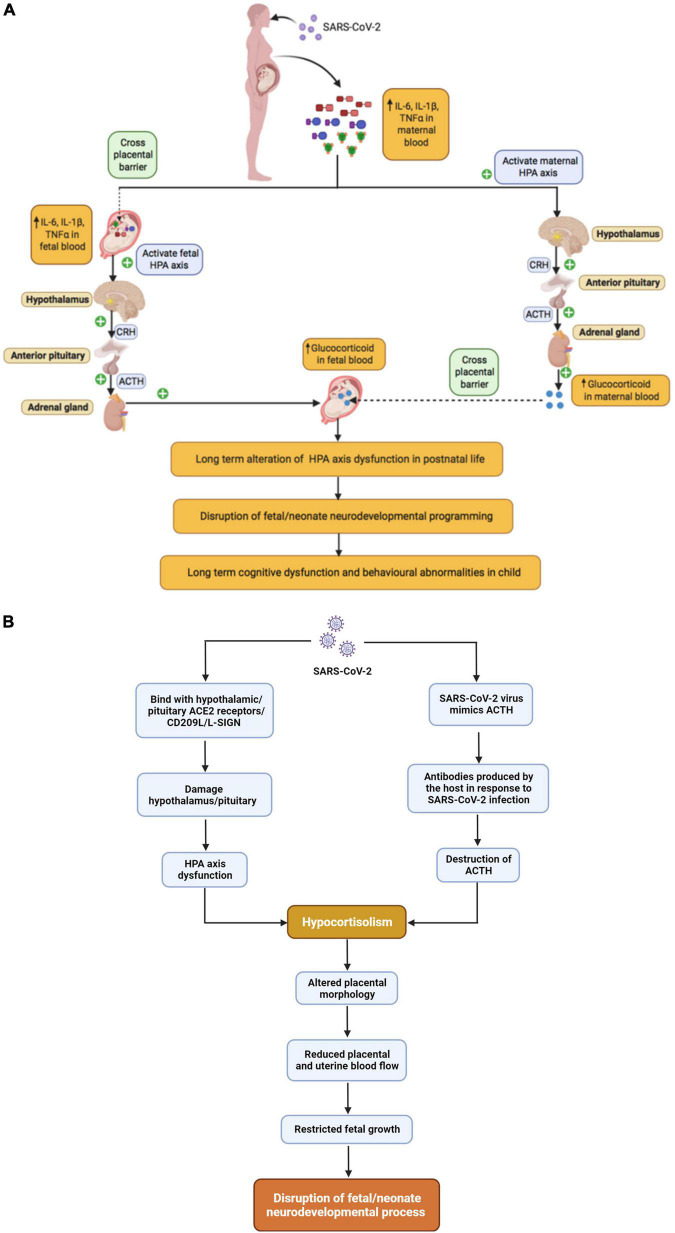
The relationship between maternal SARS-CoV-2 infection and Fetal HPA axis modulation, **(A)** SARS-CoV-2 infection and hypercortisolism, **(B)** SARS-CoV-2 infection and hypocortisolism.

It is reasonable to hypothesize that MIA in the context of SARS-CoV-2 would produce similar effects on the HPA axis which may increase risk for behavioral problems in offspring as shown in Figure 4A. Indeed, SARS-CoV-2 infected patients may experience a ‘cytokine storm’ leading to excessive glucocorticoids that may have deleterious effects on the host (Figure 4A) (Silverman et al., 2005; Raony et al., 2020). On the other hand, there is also evidence to suggest that SARS-associated coronaviruses can produce hypocortisolism (Figure 4B) (Leow et al., 2005). In a prospective cohort study of SARS-CoV survivors, 24 of 61 patients developed HPA axis dysfunction resulting in reduced blood cortisol levels during the 3-month follow-up period (Leow et al., 2005). A published case report suggests that SARS-CoV-2 infection can also produce hypocortisolism; a 69-year-old Iranian man, admitted to the ICU with SARS-CoV-2, developed adrenal insufficiency and reduced serum cortisol level (Heidarpour et al., 2020). Two mechanisms have been proposed for the association of SARS-associated coronaviruses with hypocortisolism: (1) destruction of ACTH due to infection and (2) damage to the hypothalamus. Regarding the first potential mechanism, in 2004, Wheatland proposed the molecular mimicry theory of ACTH in SARS. This theory is based on the observation that SARS-CoV expresses certain amino acid sequences that mimic the adrenocorticotropic hormone (ACTH). Thus, antibodies produced by the host in response to SARS-CoV may also destroy host ACTH thereby reducing the patient’s cortisol level (Wheatland, 2004). Regarding the second potential mechanism, the authors of the prospective cohort study of SARS-CoV survivors discussed above proposed in their discussion that SARS-CoV-2 infection could damage the hypothalamus and/or pituitary via the ACE2 receptor or CD209L/L-SIGN, a C-type lectin surface glycoprotein implicated in viral pathogenesis, leading to HPA axis dysfunction and hypocortisolism (Leow et al., 2005). Support for this mechanism has recently been provided by a French group, who reported that ACE2 and the transmembrane proteinase, serine 2 (TMPRSS2), which cleaves the SARS-CoV-2 spike protein, are expressed in the adult human hypothalamus, with the paraventricular nucleus showing the highest expression among hypothalamic nuclei. Interestingly, a KEGG pathway enrichment analysis suggested that both ACE2 and TMPRSS2 play important roles in the “neuroactive ligand-receptor interaction” pathway supporting an impact of SARS-CoV-2 on neuroendocrine function, including interactions between corticotropin releasing hormone and its receptor. Furthermore, they report that viral markers for SARS-CoV-2 were abundant in the hypothalamus of a 63-year-old male patient who died of COVID, but absent from the hypothalamus of controls (Nampoothiri et al., 2020). Very little work has been done on the possible neurodevelopmental consequences of low cortisol levels during pregnancy, but pregnancy is typically accompanied by substantial increases in cortisol (Mastorakos and Ilias, 2003; Jensen et al., 2011; Guardino et al., 2016). Several studies, conducted in sheep, indicate that lowering maternal cortisol during pregnancy alters placental morphology and reduces placental and uterine blood flow, which could result in restricted fetal growth (Jensen et al., 2005, 2007) and altered neurodevelopment, as previously discussed. Overall, depending on the timing of SARS-CoV-2 infection, the developing fetus may be exposed to both abnormally high and abnormally low levels of cortisol with potential consequences for neurodevelopment.

## Maternal infection and epigenetic modulation

Accumulating evidence suggests that maternal infection during gestation may affect intergenerational and transgenerational offspring neurodevelopmental process via epigenetic modifications (reviewed in Kleeman et al., 2022). Epigenetic processes produce long term and heritable modifications in gene expression without changing the DNA sequence (reviewed in Bale, 2015). These processes include DNA methylation, histone modification, and expression of microRNA (miRNA) (reviewed in Bale, 2015; Szyf, 2015; Weber-Stadlbauer, 2017). Epigenetic processes play a critical role in linking early environmental experiences to long-term changes in brains structure and function and are likely to play a key role in explaining the impact of maternal infection on brain development as described in subsequent paragraphs (reviewed in Bale, 2015; Dubey et al., 2018; Bergdolt and Dunaevsky, 2019).

DNA methylation involves the attachment of a methyl (CH3) group to cytosines within the DNA sequence, a reaction that requires both methyltransferases, such as DNA cytosine-5-methyltransferase 1 (DNMT1), and methyl donors, which are derived from nutrients such as folate. Methylated DNA attracts methyl binding proteins, such as methyl CpG binding protein 2 (MeCP2), that condense the structure of the nucleosome, thereby preventing transcription. A growing body of research suggests that infection during pregnancy alters DNA methylation in the offspring brain in ways that are both complex and region-specific (Richetto et al., 2017b). Richetto and colleagues observed that MIA following treatment with the viral mimetic Poly(I:C) altered DNA methylation in the medial prefrontal cortex of adult offspring. Adult offspring of immune activated mothers showed hyper or hypomethylation of CpGs at various loci including loci influencing GABAergic differentiation and signaling (*Dlx1, Lhx5, Lhx8*), Wnt signaling (*Wnt3, Wnt7b, Wnt8a*), neural development (*Efnb3, Mid1, Nlgn1, Nrxn2*) (Richetto et al., 2017b), and myelination related gene α-myelin-associated oligodendrocytic basic protein (*mobp*) in prefrontal cortex and nucleus accumbens of adult offspring (Richetto et al., 2017a), suggesting that epigenetic modification might mediate the impact of prenatal infections on offspring behavior (Richetto et al., 2017a,b). Labouesse and colleagues have also investigated Poly(I:C) induced MIA impacts on prefrontal cortex in mice and found increased methylation of promotors for GAD1 and GAD2, key enzymes in GABA synthesis (Labouesse et al., 2015). Poly(I:C) induced MIA also induces significant global DNA hypomethylation in the hippocampus, including in the promotor of *Mecp2*, but not in the mice striatum (Basil et al., 2014), as well as increased expression of DNase I hypersensitivity sites (DHSs) and MECP2 binding sites genes, namely *Abat* and *Gnas9* in mice hypothalamus (Basil et al., 2018). In another study, poly(I:C) induced MIA increased methylation of the promoter region of tyrosine hydroxylase (*Th*) gene in the dopaminergic neurons of ventral midbrain in adult mice (Weber-Stadlbauer et al., 2021). All the above-mentioned studies used Poly (I:C) induced MIA model, because it is widely applied to study neurodevelopment (reviewed in Bao et al., 2022). Further, Poly (I:C) induced MIA are mainly driven via IL-6 activity (reviewed in Bao et al., 2022). IL-6 may provide a mechanistic link between infection and altered DNA methylation as it promotes nuclear translocation of DNMT1, the major enzyme responsible for maintaining methylation patterns following DNA replication (Hodge et al., 2007). Further, alterated or defective DNA replication may lead to impaired neuronal development (Kalogeropoulou et al., 2019).

Histone modifications are post translational modifications of histone protein (Szyf, 2015). There can be hundreds of modifications on a given histone producing a cumulative effect on how DNA around that histone is packaged. The addition or removal of acetyl groups is one biochemical process known to be important for transcriptional regulation and is accomplished by two types of enzymes: histone acetyltransferases (HATs) and histone deacetylases (HDACs). HATs promote DNA histone acetylation, which loosens the chromatin and facilitates gene transcription, while HDACs remove acetyl groups, which condenses the chromatin and reduces gene transcription (reviewed in Dubey et al., 2018). LPS-induced inflammation increases expression of HDAC2 and HDAC5 in the brain, an effect that can be attenuated by treating with an HDAC inhibitor prior to administering LPS. Moreover, pre-treatment with an HDAC inhibitor diminished LPS-induced anhedonia, anorexia, and microglia activation, supporting histone deacetylation as a key mechanism linking systemic inflammation to cognitive dysfunction in adult and juvenile animals (Hsing et al., 2015). Another study in mice suggests that MIA [Poly(I:C)] regulates hippocampal serotonin transporter (SERT) levels via modulation of histone acetylation which results in anhedonic behavior in offspring (Reisinger et al., 2016). Specific cytokines linked to histone modification include TNF-alpha, which increases histone acetylation in human alveolar epithelial cells (Rahman et al., 2002) and IL-17a, which reduces HDAC2 activity via the PI3K pathway in human bronchial epithelial cells (Zijlstra et al., 2012). Whether these cytokines have similar effects in the developing brain is currently unknown. In one study, MIA [Poly(I:C)] during pregnancy did not appear to alter histone modification in cerebral cortex of adult offspring, although activation of cytokine signaling in primary cultures from fetal forebrain influenced trimethylated histone H3-lysine 4 (H3K4me3) marks in a limited set of genes (Connor et al., 2012). In contrast, Tang et al. (2013), observed that MIA [Poly(I:C)] leads to global hypoacetylation of histone H3 at H3K9K14 and H4K8 in the cortex of juvenile mice. This was accompanied by reduced expression of genes involved in neuronal development, synaptic transmission, and immune signaling. Specific genes exhibiting hypoacetylation included *Robo1*, which is involved in axon guidance and neuronal precursor cell migration, *arhgap18*, and *Ntrk3*, which is likely involved with cell survival and differentiation in the nervous system. Hyperacetylation was also observed at specific loci in the hippocampus of juvenile mice including *Disc1*, which is involved in many aspects of nervous system development, *Nr2f1*, *Ntrk3*, which is a transcriptional regulator, and *Gria1* and *Gria2*, which are both subunits of AMPA-type ionotropic glutamate receptors (Tang et al., 2013). Further, glutamate activities may modulate the early brain developmental process (Tanaka, 2013). Another, mouse study suggested that prenatal poly (I:C) exposure at late gestation (embryonic day 17) leads to deficits in working memory of the adult animal due to altered histone H3K4me3 methylation in approx. 30 genes, including Disc1 (Connor et al., 2012).

MicroRNA (miRNA) are small endogenous non-coding RNAs involved in post-transcriptional gene regulation (Ha and Kim, 2014; Weber-Stadlbauer, 2017). They target most protein coding transcription and prevent the production of specific proteins by binding to and destroying messenger RNA (mRNA). miRNA are highly expressed in brain and essential for brain development and neuronal function (Petri et al., 2014). Several recent studies demonstrate an impact of MIA on miRNA expression in offspring brain. Sunwoo et al. (2018) have reported that MIA [Poly(I:C)] alters brain miRNA expression in offspring at 3 weeks of age, a time when both synaptogenesis and myelination are ongoing. 8 miRNA were upregulated and 21 were downregulated. Furthermore, target genes of 18 downregulated and 3 upregulated miRNA were found to be significantly enriched among differentially expressed genes, confirming that MIA induced alterations in miRNA have functional consequences, at least at the level of gene expression. Offspring exhibited behavioral changes typical of MIA including lack of a preference for social novelty and reduced prepulse inhibition, but the study design did not address whether miRNA alterations played a causal role in the behavioral abnormalities (Sunwoo et al., 2018). Berger et al. examined how MIA [Poly(I:C)] affected 13 specific miRNA in offspring hippocampus and observed increased levels of miR-15b-2, miR-98-1, miR-103-2, and miR-124-1. None of these overlap with the differentially expressed miRNA in Sunwoo et al., indicating that additional studies are needed to clarify which miRNA are most robustly associated with MIA. Interestingly, MIA also influenced MiRNA expression in F2 generations of offspring, along the paternal line. The specific miRNA involved differed from observations in the F1 generation. This and the modest magnitude of changes led the authors to conclude that miRNA did not play a substantial role in the behavioral impacts of MIA (Berger et al., 2018).

With specific regard to SARS-CoV-2, no studies have examined miRNA levels in the brains of exposed fetuses. However, a recently published cohort study indicated that various miRNAs are upregulated in both plasma and placental tissues of pregnant mothers infected with SARS-CoV-2 (Saulle et al., 2021). 35 miRNA were differentially expressed in human plasma including seven antiviral miRNAs (miR-21, miR-23b, miR-28, miR-29a, miR-29c, miR-98 and miR-326) and six immunomodulatory miRNAs (miR-17, miR-92, miR-146, miR-150, miR-155, miR-223), all upregulated in infected mothers. In placenta, eight miRNA were upregulated in the context of maternal infection including ones with direct effects on viral replication (miR-21b, miR-29c, miR-98) and ones influencing viral replication by indirect mechanisms (miR-146, miR-155, miR-190, miR-346, and miR-326) (Saulle et al., 2021). Further, an *in silico* study showed that miR-21, miR-16 and miR-146a have high affinity to the SARS-CoV-2 virus (Jafarinejad-Farsangi et al., 2020).

Interestingly, several of these miRNAs have also been implicated in neurodevelopment. miR-146a is one of the most commonly dysregulated miRNAs in neurodevelopmental disorders (Fregeac et al., 2016; Nguyen et al., 2016, 2018; Schepici et al., 2019). In H9 human neural stem cells, miR-146a overexpression enhances neurite outgrowth and branching and favors differentiation into neuronal like cells (Nguyen et al., 2018). In mouse primary cell cultures, miR-146a overexpression leads to impaired neuronal dendritic arborization and increased astrocyte glutamate uptake capacities (Nguyen et al., 2016). miR-146a is highly expressed in mouse hippocampus, amygdala and entorhinal cortex, areas with important roles in social cognition, memory, spatial navigation, and the perception of time, and targets genes with known roles in neurodevelopment including *MAP1B, FMR1*, and *KCNK2* (Nguyen et al., 2016). Furthermore, miR-146a overexpression promotes oligodendrocyte differentiation and myelination in the context of neurological injury (Santra et al., 2014; Liu et al., 2017; Zhang et al., 2017, 2019), raising the possibility that it also effects these processes during neurodevelopment. In addition, downregulation of miRNA-146a expression in mice leads to impaired neurogenesis, abnormal brain anatomy, along with deficits in working and spatial memory (Fregeac et al., 2020). Apart from miR-146a, miR-21, miR-146b, miR-23a, miR-23b, miR-92(a1-a2) and miR-23a-3p have been reported to differentiate between controls and individuals with ASD in peripheral tissues, e.g., lymphoblastoid cell lines and saliva (Talebizadeh et al., 2008; Sehovic et al., 2020; Frye et al., 2021), while miR-21-3p is overexpressed in the cortex of post-mortem ASD patients (Wu et al., 2016). These miRNAs have been less thoroughly studied than MiR-146a in terms of their roles in neurodevelopment, but miR-21 expression in the placenta is associated with fetal growth (Maccani et al., 2011), which could explain the association of maternal SARS-CoV-2 infection with intrauterine growth restriction, a known risk factor for altered neurodevelopment. miR-23 regulates progenitor fate decisions by inhibiting cyclin D1 mRNA. Inhibition of miR-23 increases cyclin D1 protein in mouse progenitor cells leading to reduced neuronal differentiation during cortical neurogenesis (Ghosh et al., 2014). miR-21-3p downregulates multiple genes in a specific gene co-expression module enriched for ASD risk genes in post-mortem human brain (Wu et al., 2016). This module is upregulated in early cortical development and is enriched for genes implicated in neural development and synaptic function (Parikshak et al., 2013). In addition, miR-21-3p over-expression led to a pronounced decrease in the *PCDH19* gene (Wu et al., 2016), which encodes a cell-adhesion protein primarily expressed in the brain. Mutations in *PCDH19* are associated with both epilepsy and ASD (Redies et al., 2012).

**FIGURE 5:**
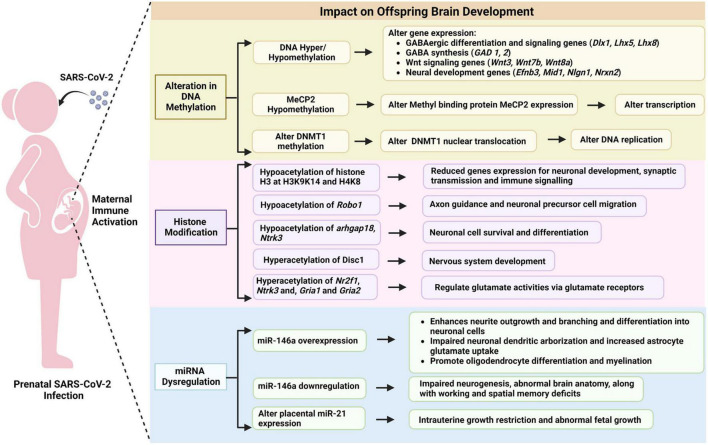
Potential epigenetic mechanisms linking prenatal SARS-CoV-2 infection to offspring brain development.

Overall, maternal immune activation can affect multiple epigenetic processes in the developing brain leading to long-lasting behavioral changes in offspring as summarized in Figure 5. Readers interested in additional details may find the following reviews of interest: (Woods et al., 2021; Kleeman et al., 2022). The latter provides a systematic review of MIA-induced changes in gene expression and epigenetic features. Additional research is needed to study these relationships in the context of maternal SARS-CoV-2.

## Emerging evidence linking gestational SARS-CoV-2 infection to altered neurodevelopment

The information presented thus far strongly suggests that gestational SARS-CoV-2 infection could alter fetal neurodevelopment. Empirical evidence supporting this hypothesis is currently sparse and somewhat inconsistent, but intriguing. A preliminary study conducted in 2020 (*N* = 57) reported that a substantial proportion of children born to mothers infected with SARS-CoV-2 during gestation were identified as high risk for social and emotional problems at 3 months of age using the Ages and Stages Questionnaire: Social-Emotional, second edition (ASQ:SE-2) (63.6%) (Wang et al., 2020). In contrast, the proportion of children identified as high risk using the Ages and Stages Questionnaires, third edition (ASQ-3) was lower – 0% for communication and gross motor skills, 5.8% for fine motor skills and problem solving, and 9.6% for personal-social skills. No control group was included for comparison. Interestingly, gross motor, problem solving, personal–social, and social–emotional were negatively linked with the amount of time mothers and babies were separated after birth. More recent studies with larger sample sizes provide additional insights (Aldrete-Cortez et al., 2022; Ayed et al., 2022; Edlow et al., 2022). A prospective cohort study conducted in Kuwait (*N* = 298) reported developmental delays in around 10% of infants whose mothers had COVID-19 during pregnancy using the ASQ-3 (Ayed et al., 2022), which is similar to rates of developmental delay in healthy children in a similar geographical and cultural setting (Charafeddine et al., 2019). The ASQ:SE-2 was not included in this study. Risk of developmental delay was significantly higher in infants born to mothers infected during the first and second trimester than mothers infected in the third trimester, suggesting that adverse neurodevelopmental consequences of SARS-CoV-2 may be time-specific (Ayed et al., 2022). Key neurodevelopmental events occurring in the first and second trimesters include formation of the neural tube and neurogenesis (Stiles and Jernigan, 2010). A study of 254 infants born in New York City during the pandemic (114 exposed to COVID-19 *in utero* and 141 unexposed) and 62 infants born before the pandemic, revealed that birth during the pandemic was associated with significantly lower scores on gross motor, fine motor, and personal-social skills, with no significant differences between the exposed and unexposed groups (Shuffrey et al., 2022). This suggests that COVID-19-related stress may have a stronger impact on neurodevelopment than gestational exposure to SARS-CoV-2. Relatively few women had confirmed exposures in early pregnancy, but *post hoc* analyses suggested exposure in the first trimester might have adverse neurodevelopmental consequences, similar to the earlier Ayed study. The above studies all relied on the ASQ-3, a parent-response instrument used to screen for developmental delays, as an index of early neurodevelopment. Aldrete-Cortez et al. took a different approach using observations of early motor repertoires as their outcome. They showed that at 3-5 months of age, a motor optimality score was significantly lower in infants prenatally exposed to SARS-CoV-2 than unexposed controls, suggesting they are at higher risk for later neurological disorders (Aldrete-Cortez et al., 2022). Finally, Edlow et al. used electronic health records and a retrospective cohort design to test associations between SARS-CoV-2 exposure *in utero* and risk for neurodevelopmental disorders in the first year of offspring life (Edlow et al., 2022). This cohort included 7772 live births of which 222 were to SARS-Cov-2 positive mothers. Maternal SARS-CoV-2 positivity during pregnancy was associated with greater rate of neurodevelopmental diagnoses in both unadjusted models and models adjusted for race, ethnicity, insurance status, offspring sex, maternal age, and preterm status. In contrast to studies using the ASQ-3, third-trimester exposures appeared to confer greater risk. Finally, a recent meta-analysis suggested that infants born during the SARS-CoV-2 pandemic had similar rates of neurodevelopmental issues compared to those born before the pandemic, regardless of whether they were exposed *in utero*. However, communication impairments were more common in children born during the pandemic and fine motor deficits were more common in those with *in utero* exposure (Hessami et al., 2022). Overall, emerging evidence suggests maternal SARS-CoV-2 infection may impact neurodevelopment but is subject to several limitations. Sample sizes are relatively small, follow-up has been limited to the first year of life, and most studies have relied on a single parent-report instrument designed to screen for developmental delays. Thus, there is an urgent need for long term follow up in infants born during the pandemic.

## Conclusion

Infection of pregnant women with SARS-CoV-2 could influence fetal brain development, potentially increasing risk for later cognitive and behavioral problems. SARS-CoV-2 infection can be transferred transplacentally from an infected mother to her fetus and the virus does have neuroinvasive potential. However, this appears to be a relatively rare event. Consequently, we anticipate that long-term neurodevelopmental effects, if they occur, are more likely to reflect indirect mechanisms. Like other viruses, SARS-CoV-2 infection may trigger maternal immune activation, which may disrupt fetal neurodevelopmental and lead to long term cognitive and motor deficits, behavioral abnormalities, and, potentially, psychiatric illness in children. SARS-CoV-2 may also trigger preeclampsia, preterm birth, and/or intrauterine growth restriction, which are known risk factors for later neurodevelopmental issues. Key finding related to SARS-CoV-2 infection are summarized in Table 1. Given the high numbers of pregnant women that have been, and will be, exposed during this pandemic, the long-term impact of SARS-CoV-2 on fetal brain development needs to be investigated.

**TABLE 1:** Effect of maternal SARS-CoV-2 infection on offspring neurodevelopment.

**Transmission of SARS-CoV-2 mother to fetus**	
SARS-CoV-2 virus infects fetus from direct transmission from infected mother to fetus in uterus	Dong et al., 2020; Kirtsman et al., 2020; Patanè et al., 2020; Sisman et al., 2020
No or very less chances of SARS-CoV-2 to vertical transmission from mother to fetus in uterus	Khan et al., 2020; Knight et al., 2020; Xiong et al., 2020; Zhu et al., 2020; Allotey et al., 2022
Neuroinvasiveness of SARS-CoV to human brain *via* binding to the endothelial ACE2 receptors of the blood brain barrier and subsequently entry into the central nervous system	Paniz-Mondolfi et al., 2020
**Time dependent inflammatory response during pregnancy against SARS-CoV-2 infection**	
Robust inflammatory responses in placenta tissue from third trimester COVID-19 infections including increased expression of pro-inflammatory genes and chemokines	Lu-Culligan et al., 2021
In decidual tissue from individuals with third trimester COVID-19 infections have increased macrophages, NK cells, and T cells, but reduced IL-8 levels and no variation in IFN-γ, IL-1β, IL-6, IL-10, and TNF levels	Juttukonda et al., 2022
In decidual tissue from individuals with second trimester infections showed a significant decrease in IL-6, IL-8, IL-10, and TNF-α and no change in abundance for IL-1β or IFN-γ	Juttukonda et al., 2022
Gender based differences, increased levels of IFN-α, IFN-γ, and IL-10 in placentas from individuals with third trimester infections, but only in males	Bordt et al., 2021
**Effect of prenatal infection of SARS-CoV-2 on fetuses and infants**	
Infants prenatally infected with SARS-CoV-2, diagnosed with neurodevelopmental disorders mainly related with motor function or speech and language disorders	Edlow et al., 2022
Children born to mothers infected with SARS-CoV-2 during gestation were identified as high risk for social and emotional problems	Wang et al., 2020
Infant born during the pandemic associated with significantly lower scores on gross motor, fine motor, and personal-social skills, with no significant differences between the exposed and unexposed groups.	Shuffrey et al., 2022
Infants born during the SARS-CoV-2 pandemic had similar rates of neurodevelopmental issues compared to those born before the pandemic, regardless of whether they were exposed *in utero*. Communication impairments were more common in children born during the pandemic and fine motor deficits were more common in those with *in utero* exposure	Hessami et al., 2022
Infants prenatally exposed to SARS-CoV-2 have low motor activity score and higher risk for later neurological disorders	Aldrete-Cortez et al., 2022
10% of infants whose mothers had COVID-19 during pregnancy exhibited developmental delays, which is similar to rates of developmental delay in healthy children in a similar geographical and cultural setting	Ayed et al., 2022

## Author contributions

HD conceived the idea for the manuscript and wrote the first draft. RS, SK, and RK contributed to manuscript revision. All authors read and approved the submitted version.
